# Synthesis of a thermoresponsive crosslinked MEO_2_MA polymer coating on microclusters of iron oxide nanoparticles

**DOI:** 10.1038/s41598-021-83608-z

**Published:** 2021-02-17

**Authors:** Alejandro Lapresta-Fernández, Alfonso Salinas-Castillo, Luis Fermín Capitán-Vallvey

**Affiliations:** 1 ECsens Group, Department of Analytical Chemistry, Campus Fuentenueva, University of Granada, 18071 Granada, Spain; 2 Unit of Excellence in Chemistry Applied To Biomedicine and the Environment of the University of Granada, Granada, Spain

**Keywords:** Polymer chemistry, Nanocomposites, Polymers

## Abstract

Encapsulation of magnetic nanoparticles (MNPs) of iron (II, III) oxide (Fe_3_O_4_) with a thermopolymeric shell of a crosslinked poly(2-(2-methoxyethoxy)ethyl methacrylate) P(MEO_2_MA) is successfully developed. Magnetic aggregates of large size, around 150–200 nm are obtained during the functionalization of the iron oxide NPs with vinyl groups by using 3-butenoic acid in the presence of a water soluble azo-initiator and a surfactant, at 70 °C. These polymerizable groups provide a covalent attachment of the P(MEO_2_MA) shell on the surface of the MNPs while a crosslinked network is achieved by including tetraethylene glycol dimethacrylate in the precipitation polymerization synthesis. Temperature control is used to modulate the swelling-to-collapse transition volume until a maximum of around 21:1 ratio between the expanded: shrunk states (from 364 to 144 nm in diameter) between 9 and 49 °C. The hybrid Fe_3_O_4_@P(MEO_2_MA) microgel exhibits a lower critical solution temperature of 21.9 °C below the corresponding value for P(MEO_2_MA) (bulk, 26 °C). The MEO_2_MA coating performance in the hybrid microgel is characterized by dynamic light scattering and transmission electron microscopy. The content of preformed MNPs [up to 30.2 (wt%) vs. microgel] was established by thermogravimetric analysis while magnetic properties by vibrating sample magnetometry.

## Introduction

Nanoscale-sized particles, combining properties from different materials, offer new tools in the field of bioengineering and biomaterials. Smart polymers can be chosen to exhibit stimuli-responsive properties whereby their structure is strongly affected by the external conditions (*e.g*. light, temperature, pH, concentration of chemicals, pressure or electronic fields)^1–5^. Among them, thermoresponsive polymers have gained a great deal of attention since the phase transition between hydrophilic and hydrophobic states involves a considerable change in volume when reaching a lower critical solution temperature (LCST)^6–10^. When swelling (below the LCST), water molecules are incorporated to the polymer chains by hydrogen bonds. Above the LCST, the hydrogen bond networks between the polymer and the water molecules are disturbed, favouring hydrophobic polymer interactions (due to the high hydrophobicity of the methylene groups) and promoting mechanical polymer contractions. The combination of MNPs with thermoresponsive polymers^11–15^ leads to the formation of colloidal composites which open new perspectives in biomedicine. Applications include targeting specific sites by applying an external magnetic field, in biosamples^16^, drug delivery^17,18^ or in hyperthermia through the application of a high-frequency electromagnetic field^19^. Although the majority of the temperature-responsive polymers are based on poly(N-isopropylacrylamide) P(*N*-iPAAm)^20–22^, a very promising thermoresponsive polymer for biomedical applications is poly (2-(2-methoxyethoxy)ethyl methacrylate, P(MEO_2_MA)^15,16,23–26^. This polymer is principally composed of biocompatible oligo(ethylene glycol) units not containing acrylamides and showing similarly interesting features as poly(ethylene glycol) PEG^21,25–29^, such as higher biocompatibility, extending thus the circulation time. However, some work still has to be invested to establish the increased the biocompatibility of these coatings. Moreover, the MEO_2_MA LCST (determined to be 26 °C) can be fine-tuned up to 90 °C with longer oligo(ethylene glycol) methacrylates (OEGMA)^29–33^, and exhibits a more uniform thermal profile than P(*N*-iPAAm)^20,27^. Therefore, the advances in alternative polymers to P(*N*-iPAAm) represent clear opportunities for new advances in emerging biomedical and materials fields due to their increased biocompatibility and tuneable response^34^. Here, the confinement of preformed Fe_3_O_4_ NPs in the P(MEO_2_MA) shell is obtained by anchoring vinyl groups on the external surface of MNPs through the use of 3-butenoic acid^35,36^. The MNPs are stabilized electrostatically using a cationic surfactant such as hexadecyltrimethylammonium bromide (CTAB), which facilitates the incorporation of the butenoic acid in the nanoparticle system. In this stabilized form, butenoic acid will provide anchor points, leading to a polymerization of MEO_2_MA in the presence of CTAB^37^. The polymers precipitate at 70 °C (i.e., above the LCST) and form particles^38^. Thus, hybrid microgels are formed showing swollen and collapsed states between 9 and 49 °C and a maximum swelling-to-collapse transition volume, *i.e*. the volume ratio between the expanded and shrunk states, of 21:1.

## Experimental

### Chemicals

All chemicals used were of analytical-reagent grade and all aqueous solutions were prepared using purified water produced with a Milli-RO 12 plus Milli-Q water system (Millipore, Bedford, MA). For preparing the core–shell hybrid microgels, hexadecyltrimethylammonium bromide (CTAB), poly (2-(2-methoxyethoxy)ethyl methacrylate P(MEO_2_MA), tetraethylene glycol dimethacrylate (TEGDMA), citric acid monohydrate, disodium hydrogen phosphate dihydrate, sodium dihydrogen phosphate monohydrate, 3-butenoic acid (3-bt), 2,2′-azobis(2-methylpropionamidine) dihydrochloride (AAPH), aqueous ammonia 25% w/w in water; ferrous (FeCl_2_·4H_2_O) and ferric chlorides (FeCl_3_·6H_2_O) were purchased from Sigma-Aldrich. The monomer MEO_2_MA was purified by passing through a neutral alumina column. The rest of the chemicals were used as received.

### Characterization

The morphology of the hybrid microgels was studied by high resolution transmission electron microscopy (HRTEM) using a Philips CM20 operating at 200 kV. Dynamic light scattering (DLS) experiments as well as zeta potential measurements, were conducted using a Zetasizer nano S90 (Malvern Instruments, Malvern UK)^39,40^. The temperature range studied was between 9 and 49 °C. Each hydrodynamic diameter of the hybrid microgels was based on intensity measurements and averaged from three independent measurements and after five minutes of temperature equilibration time. The iron (II, III) oxide (Fe_3_O_4_) nanoparticle content (wt %) inside the hybrid microgels was determined by thermogravimetric analysis (TGA) performed on a TGA Mettler-Toledo mod. TGA/DSC1 at a heating rate of 10 °C min^−1^ under nitrogen. The magnetization of the coated and uncoated MNPs was measured by using a Quantum Design (SQUID) magnetometer MPMS-7 T equipped with a superconductor magnet of 7 T and a home-made vibrating sample magnetometer (VSM) operating up to 1.8 T.

### Synthesis of the hybrid (Fe_3_O_4_@P(MEO_2_MA) microgel

The iron oxide nanoparticles were synthesized according to a previously published procedure^41^. Briefly, iron oxide nanoparticles of magnetite (~ 10 nm in diameter) were formed by the co-precipitation of ferrous (0.86 g) and ferric salts (2.36 g) dissolved in water (40 mL) at 80 °C under an argon atmosphere. After 30 min of reaction, 1 g of citric acid in 2 mL of water was added dropwise and a black precipitate was collected and subjected to a magnetic decantation and cycles of centrifugation/ultrasonic redispersion in water to remove all soluble substances. The MEO_2_MA coating was prepared as described by Contreras-Cáceres et al.^22,36^ using the fact that the monomers are initially soluble in water, but the formed polymers precipitate at 70 °C (i.e., above the LCST) and form particles^38^. To incorporate vinyl functionality on the MNPs surface^35,36^, butenoic acid (50 μL) was added to the MNPs (200 μL at 5 mg·mL^−1^) and diluted in 10 mL of water during sonication (15 min, to decrease the possible formation of magnetic clusters due to particle aggregation). The dispersion was maintained at 70 °C for 30 min. After that, CTAB (0.6 mL 0.1 M) was added to increase the particle stability while decreasing the particle aggregation in the centrifugation process (5000 rpm for 30 min) which removes excess of butenoic acid. Next, the thermoresponsive coating is formed by free-radical precipitation polymerization. The pellet was heated to 70 °C under N_2_ atmosphere (having previously been purged for 10 min) followed by the addition of MEO_2_MA (0.1164 g, 0.618 mmol) and TEGDMA (0.0204 g, 0.062 mmol, 10% content with respect to MEO_2_MA). After 15 min of mechanically stirring under N_2_ flow, the polymerization was started by adding AAPH (100 μL, 0.1 M). Approximately 3 min later, the solution became cloudy, indicating that polymerization started, and was allowed to proceed for 2 h at 70 °C. Finally, the light brown product was cooled down to room temperature and purified with four cycles of magnetic separation with a neodymium magnet followed by probe type ultrasonic redispersion in water (15 mL). To exclude aggregates of higher dimensions, the hybrid microgels were filtered through a 0.45 µm syringe filter (Whatman, Dassel, Germany).

## Results and discussion

In this study, a modified coating method based on 3-butenoic acid to promote vinyl functionality on the MNPs surface was used to promote the successful PMEO_2_MA coating^35^. After that, the encapsulation of the magnetic cores was carried out by removing the excess of butenoic acid with CTAB and mixing the hydrophilic monomer MEO_2_MA and the crosslinker TEGDMA (10%) in the presence of a water soluble cationic azo-initiator (AAPH) at 70 °C^37,42^.

**Scheme 1:**
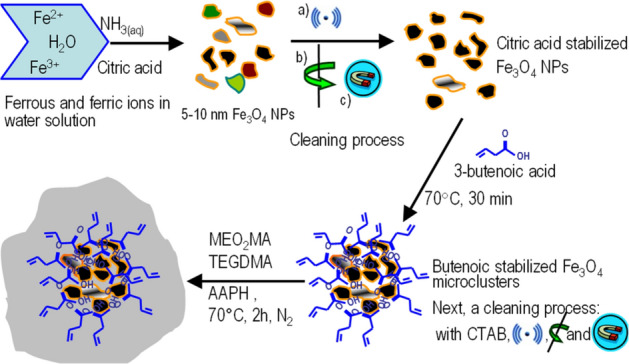
End to end preparation of the hybrid Fe_3_O_4_@MEO_2_MA microgel: Surface modification with 3-bt to develop the P(MEO_2_MA) coating; cleaning process including (**a**) ultrasonic redispersion (**b**) centrifugation and (**c**) magnetic decantation.

**Figure 1:**
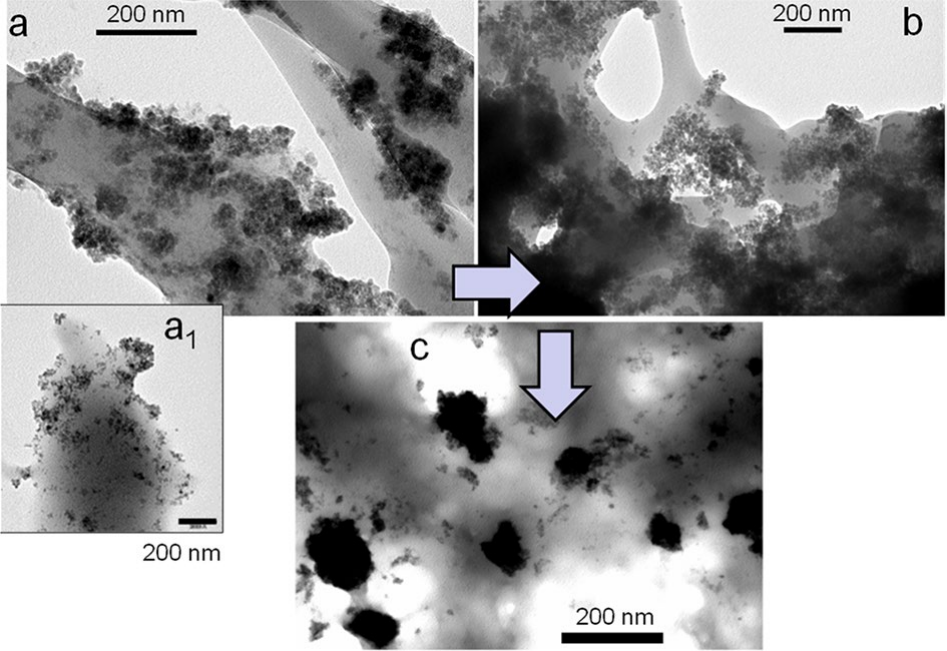
TEM image of the polymerization under different conditions: (**a**-**a**_**1**_) in absence of 3-bt: MNPs are deposited mostly on the surface of the thermoresponsive polymer, (**b**) with 3-bt to promote a terminal double bond over the NPs surface: Large aggregates of MNPs are embedded in the polymer matrix, and (**c**) using 3-bt and CTAB at room temperature: well defined microclusters of MNPs are obtained.

Scheme 1 summarizes the synthesis procedure of the hybrid microgel, which is a modification of a previously reported method^35,36^. In the optimization procedure some key steps have to be considered such as the addition of 3-bt and CTAB and the temperature. Thus, in the absence of both 3-bt and the CTAB at 70 °C, MNPs were heterogeneously loaded on the surface of a bulky hydrogel (micro-sized hydrogels were not formed) being preferentially localized at the periphery of the crosslinked polymeric material (Fig. 1a,a1). That is, interestingly, the MNP are coating the polymer, forming a thermoresponsive-magnetic composite, P(ME_2_OMA)@Fe_3_O_4_. This phenomenon can be attributed to the attractive forces between the negatively charged MNPs (coated with citric acid, ξ potential about − 35 mV) and the positive charge found in the synthesis bath (ξ potential measurements during the synthesis changed from − 35 mV (citric acid MNPs) to + 15 mV (end of the synthesis)).

This can be explained by the positive charge density due to the presence of the cationic azo-initiator AAPH, which would lead to an increased surface interaction between the polymer and the MNPs. These interactions could arise from electrostatic attraction and lead to the formation of composites of polymer coated with MNPs that exhibit low colloidal stability which would then sediment rapidly.

Conversely, when 3-bt is added to the system without heating to 70 °C (i.e. at room temperature), large composites are formed with the MNPs embedded inside the polymeric matrix in a heterogeneous composite (Fig. 1b). The vinyl functionality from the 3-bt allows the MNPs to be embedded into the thermoresponsive hydrogel. When the polymerization is developed with MNPs, 3-bt and CTAB at room temperature, the MNPs are grouped inside the polymeric matrix involving the formation of magnetic microclusters embedded inside the microgel with relatively homogeneous sizes between 100–200 nm (See Fig. 1c). Consequently it is likely that the effect of the 3-butenoic acid is to promote the polymerization process on the surface of the MNPs hence enabling the MNPs to be embedded inside the polymeric matrix (Fig. 1b), while electrostatic forces are responsible of the coating of MNPs on the polymeric surface (P(MEO_2_MA)@ Fe_3_O_4_) (Fig. 1a-a1). Moreover, the effect of CTAB in this system is to stabilize the modified MNPs with 3-bt into larger entities. Similarly to a previously work based on gold NPs as cores^37^, the CTAB could coat the MNPs with a thick hydrophobic bilayer, allowing the confinement of the 3-bt acid within the layer and anchoring the vinyl groups on the external surface of the MNPs. Under these conditions, large composite materials are formed in a non-colloidal state. However, at 70 °C the hybrid (Fe_3_O_4_@P(MEO_2_MA) microgels are developed in a precipitation polymerization process where the polymer is insoluble and precipitates as explained below.

### Thermoresponsive P(MEO_2_MA) coating

**Figure 2:**
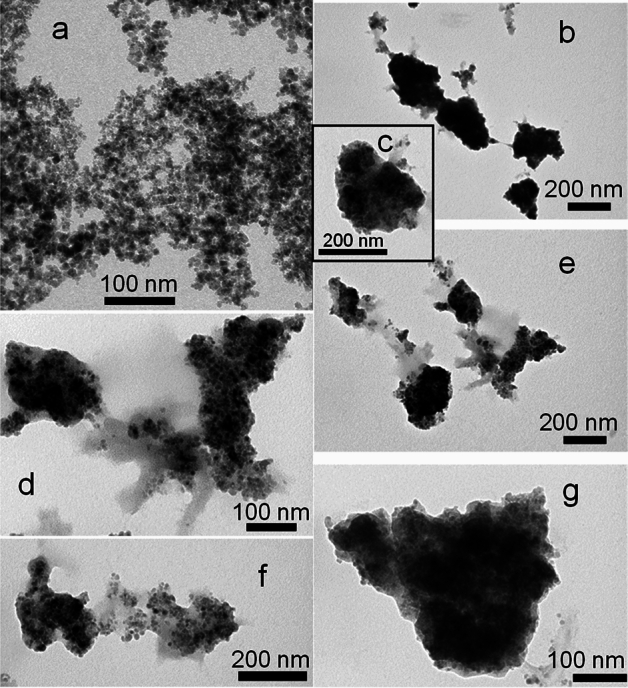
TEM images of (**a**) MNPs of diameter around 5–10 nm. From (**b**) to (**g**) hybrid (Fe_3_O_4_@P(MEO_2_MA) microgels, particle diameter around 150–200 nm.

By increasing the temperature to 70 °C, the 3-bt will serve as anchor points providing the MNPs with vinyl functionality, so that they become, by themselves, "polymerization macroinitiators" of the polymerization of MEO_2_MA in the presence of CTAB^37^. Under these conditions, thermoresponsive and magnetic microgels can be formed as the polymers precipitate at 70 °C (i.e., above the LCST) and form particles (Fig. 2)^38^. The diameter of such hybrid microgels is around 150–200 nm (Fig. 2b,g). Figure 2a displays TEM images of the Fe_3_O_4_ NPs with diameters around 5–10 nm before the polymer coating. The thermoresponsive properties of these core–shell microstructures were characterized by DLS^39^. When applying a change in the local environment, temperature in this case, an environmentally-initiated phase transition is observed in the hybrid microgels. At lower temperatures the hydrophilicity behaviour (polymer chains forming hydrogen bonds with water molecules) is predominant and the polymer is swollen while at higher temperatures the hydrophobic tendency (bound water molecules are transported out of the polymer region) is predominant and the polymer chains shrink into a crosslinked structure.

**Figure 3:**
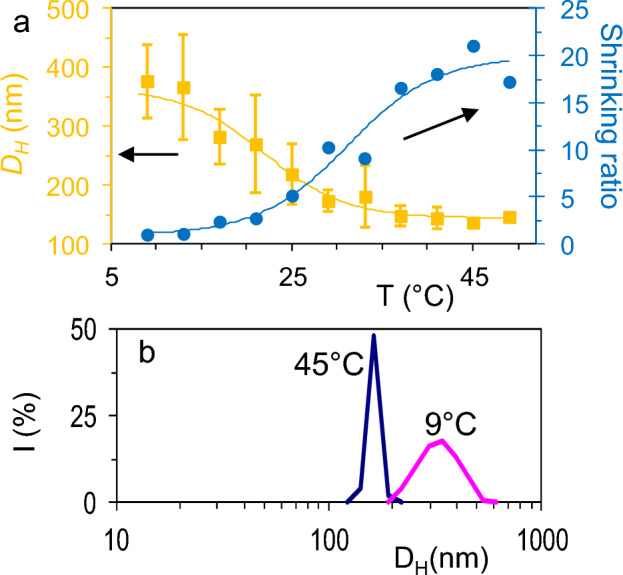
(**a**) Temperature dependence of D_H_ for hybrid Fe_3_O_4_@PMEO_2_MA microgels and the shrinking ratio (heating from 9 to 49 ºC), (**b**) DLS measurements based on the scattered intensity of the hybrid microgels measured at 9 °C and 45 °C. Errors bars indicate the standard deviation of three replicates each.

The variation of the hydrodynamic diameter (D_H_) of the hybrid microgels when the temperature is varied between 9 and 49 °C, follows a sigmoidal tendency which can be adjusted to a sigmoidal fit using a Boltzmann type equation (Eq. 1). From this fit, a well-defined volume phase-transition temperature around 21.9 °C is obtained. (Fig. 3a).

1 $$D_{{\text{H}}} (nm) = a_{{2(collapsed)}} + \frac{{a_{{1(Swollen)}} - a_{{2(Collapsed)}} }}{{1 + \exp \left( {\frac{{\left( {T - a_{{o(LCST)}} } \right)}}{{a_{3} }}} \right)}}$$

where a_0_ to a_3_ are adjustable coefficients: a_1_ and a_2_ are the hydrodynamic diameters in the collapsed and in the swollen state, respectively while a_0_ is the midpoint transition temperature which is related to the LCST value (see data in Table 1)^5,43^.

**Table 1:** Hybrid Fe_3_O_4_@PMEO_2_MA microgel parameters.

Parameters	Value
MEO_2_MA (mol)	6.01 · 10^–4^
TEGDMA (mol%)^a^	10
Swollen state diameter (nm)	364.0
Collapsed state diameter (nm)	143.7
LCST (°C)^b^	21.9
Shell thickness increment (nm)	220.3
Shrinking volume	21.1
Slope (°C^−1^)^c^	12.1
MNPs content (wt%)	~ 26.9
Saturation magnetization (A m^2^ Kg^−1^)	14.10

^a^Percentage with respect to the molar content of MEO_2_MA. ^b^ LCST calculated with the central point of the Boltzmann fit. ^c^Absolute value.

As the temperature is changed (heating from 9 to 49 °C), we observe a change in the average diameter from 143.7 nm for the hybrid microgels in the collapsed state to approximately 364.0 nm in the swollen state. The transition from the swollen to the collapsed state proceeds rapidly as shown by the high value of the slope of the sigmoidal fit in Fig. 3a (12.1 °C^−1^). The slope value was obtained by fitting the central part of the sigmoidal function, to a linear relationship by means of a lack-of-fit test (see Fig. 3a and Table 1).

The shrinking ratio^42^ defined as the ratio between the volume of the particle in the fully swollen state (at 9 °C) and the volume of the particle at each temperature (β = V_swollen_(9 °C)/V(T)), shows an increase of approximately 21 times, as shown in Fig. 3a (right axis). As expected, the size distribution for the hybrid microgels (Fig. 3b) in the swollen state (9 °C) is larger than that of the collapsed state (49 °C), due to the random coil configuration of the polymer at low temperatures. When decreasing the temperature, the hydrophilic behaviour of the MEO_2_MA starts to dominate and the variability in the increasing hydrodynamic diameter is higher (being relatively uniform in the collapsed state) as shown in Fig. 3a.

The measured LCST value for the hybrid microgels (21.9 °C) is smaller than the LCST for MEO_2_MA (26 °C) which could indicate the small size of the polymer (coating the MNPs) leading to a significant decrease in LCST. On the other hand, an irreversible behaviour in the D_H_ is observed when cooling from 49 to 1°C. Thus, once the hybrid microgels reached 49 ºC (heating from 9 to 49 °C, Fig. 3a), the molecular structure is disturbed during a subsequent cooling, observing that the D_H_ did not follow any trend with temperature (Fig. S1). Hence loosing the previous relationship in D_H_ induced by thermal variation when heating.

#### Thermal properties

**Figure 4:**
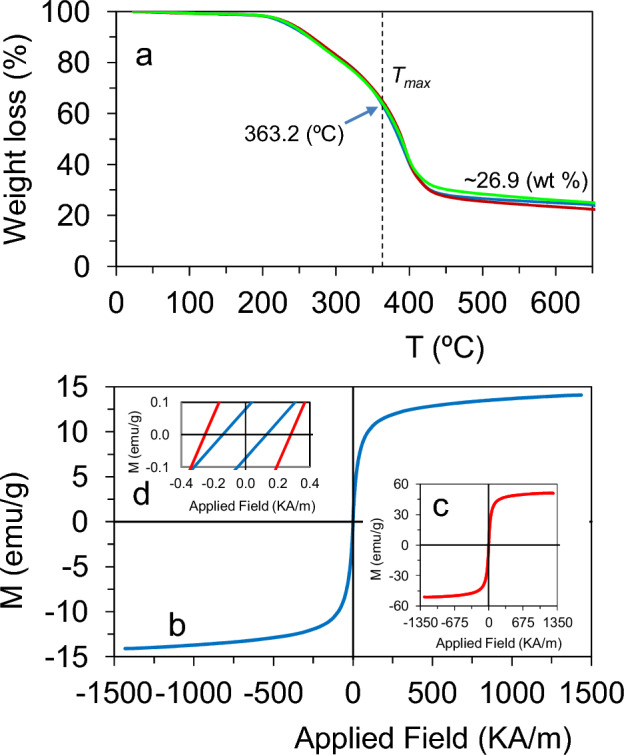
(**a**) TGA curves, in triplicate (red, blue and green curves), show the *T*_*max*_ at around 363.2 ºC and the weight loss of the hybrid microgels which gives a content of pre-formed MNPs of around 26.9 (wt %), (**b**) Field dependant magnetization curve of hybrid microgels. The inset (**c**) corresponds to the magnetization curve for the uncoated Fe_3_O_4_ NPs while the inset (**d**) is the coercivity (*Hc*) for coated and uncoated MNPs. Blue line: hybrid microgel; Red line: uncoated MNPs.

The thermal behaviour of the hybrid microgels was investigated by TGA. Figure 4a shows the TGA curves (of three different samples) demonstrating a main decomposition process at around 363.2 °C, in good agreement with the decomposition temperature (*T*_*max*_) of P(ME_2_OMA) which is placed at around 369 °C^44,45^. The weight loss of the hybrid microgels indicates a high content of preformed MNPs (up to 30.2 wt % vs. microgel) with an average value of 26.9 wt % (value obtained from three different samples).

#### Magnetic characterization

The magnetic properties for hybrid microgels were examined by VSM at room temperature. Figure 4b shows the field dependent magnetization for the magnetic nanoparticles before and after coating with PMEO_2_MA. As expected, the saturation magnetization (*M*_*s*_) for the hybrid microgels (14.10 A m^2^ Kg^−1^) was lower than that corresponding to the MNPs (50.86 A m^2^ Kg^−1^) due to the diamagnetic contribution of the polymer coating (Fig. 4b,c). This decrease is attributed to the reduced proportion of the Fe_3_O_4_ NPs compared to the total mass. Thus, the fraction of Fe_3_O_4_ NPs loaded into the hybrid microgels decreased to approximately 27.7 (wt %) of the total mass of the hybrid microgel (MNPs and polymer). This value is in good agreement with the MNPs content obtained by TGA (26.9 wt %). Moreover, the remanent magnetization (*M*_*r*_) also decreases in the hybrid microgels (from 0.300 to 0.068  A m^2^ Kg^−1^) due to the standard practice of normalizing the magnetization by sample mass. The magnetic measurements also indicate a ferrimagnetic behaviour since coercivity (*Hc*) is observed (Fig. 4d). The decrease in *H*_*c*_ upon polymeric coating from 0.266 to 0.137 KA m^−1^ could indicate that the polymeric coating may affect the contributions of the surface anisotropy and/or interparticle interactions^1,46^. It was previously reported that a dense population of MMPs decorating a polymeric microgel might increase or even eliminate the volume phase transition temperature (VPTT)^12,47,48^. Such studies reported that the introduction of a high number of MNPs could introduce steric hydrances inside the polymer matrix thereby inducing a restriction of movement imposed by the presence of MNPs and hence producing a total disappearance of the volume phase transition. In our case, it can be suggested that the magnetic clusters loaded in the hybrid microgel may also decrease the LCST as consequence of the MNP-polymer interactions that would induce a restriction of movement in the polymer resulting in a modulation of the swelling-to-collapse transition. Thus, the presence of MNPs involves different interactions (MNPs-MNPs and MNPs-polymer) that may influence the transition temperature of the hybrid microgel tuning the LCST. A similar microgel, but using a single Au nanoparticle as central core, showed full reversibility during four different cycles of cooling and heating^5^, while here an irreversible behaviour is observed when cooling down just after a heating from 9 to 49 °C. Therefore, further studies are required to establish a MNPs content-dependant LCST.

## Conclusions

The feasibility of hybrid magnetic microgels consisting of cores of MNPs (~ 5–10 nm in diameter) coated with a thermoresponsive shell of a highly biocompatible polymer, P(MEO_2_MA) has been demonstrated. The confinement of the preformed Fe_3_O_4_ NPs with P(MEO_2_MA) is mediated by a free-radical precipitation polymerization method in the presence of CTAB and 3-butenoic acid at 70 °C in aqueous media. The shrinking ratio shows a value of 21.1, indicating the high difference in volume between the collapsed and the swollen states. The hybrid microgels were loaded with approximately 30.2% of MNPs (distributed inside the volume of the microgel) and showed a LCST of 21.9 °C; lower than that observed in the MEO_2_MA bulk (26 °C). The saturation magnetization of the hybrid microgels decreased on covering the MNPs with the P(MEO_2_MA) shell (from 50.86 to 14.10 A m^2^ Kg^−1^) while the fraction of Fe_3_O_4_ NPs loaded into the hybrid microgels was in good agreement between TGA (~ 26.9 wt %) and magnetic measurements (~ 27.7 wt %). This magnetic-thermoresponsive material open interesting perspectives in applications as smart drug delivery systems where a localized temperature change is used to initiate the volume phase transition which in turn would trigger the drug release in the desired location.

### Supplementary Information

Supplementary Information
