# Author Correction: Synthesis of a thermoresponsive crosslinked MEO_2_MA polymer coating on microclusters of iron oxide nanoparticles

**DOI:** 10.1038/s41598-024-59793-y

**Published:** 2024-04-19

**Authors:** Alejandro Lapresta-Fernández, Alfonso Salinas-Castillo, Luis Fermín Capitán-Vallvey

**Affiliations:** 1 ECsens Group, Department of Analytical Chemistry, Campus Fuentenueva, University of Granada, 18071 Granada, Spain; 2 Unit of Excellence in Chemistry Applied To Biomedicine and the Environment of the University of Granada, Granada, Spain

Correction to: *Scientific Reports* 10.1038/s41598-021-83608-z, published online 17 February 2021

The Acknowledgements section in the original version of this Article was incomplete.

“A. Lapresta-Fernández acknowledges the Andalucía Talent hub Post-doc fellowship supported by the Andalusian Knowledge Agency, COFUND by the EU 7th Framework Program, Marie Skłodowska-Curie actions 2014 (Ref: Talent Hub 2014-8). (Grant Agreement nº 291780) and The Ministry of Economy, Innovation, Science and Employment of the Junta de Andalucía. This study was supported by Spanish “Ministerio de Economía y Competitividad” (Projects PID2019-103938RB-I00 and CTQ2017-86125-P) and Junta de Andalucía (Projects B-FQM-243-UGR18 and P18-RT-2961) and CEI-Biotic (CEI2013-MP-10). We also acknowledge the “Unidad de Excelencia Química Aplicada a Biomedicina y Medioambiente” (UGR).”

now reads:

“A. Lapresta-Fernández acknowledges the Andalucía Talent hub Post-doc fellowship supported by the Andalusian Knowledge Agency, COFUND by the EU 7th Framework Program, Marie Skłodowska-Curie actions 2014 (Ref: Talent Hub 2014-8). (Grant Agreement nº 291780) and The Ministry of Economy, Innovation, Science and Employment of the Junta de Andalucía. This study was supported by Spanish “Ministerio de Economía y Competitividad” (Projects PID2019-103938RB-I00 / AEI / 10.13039/501100011033 and CTQ2017-86125-P) and Junta de Andalucía (Projects B-FQM-243-UGR18 and P18-RT-2961) and CEI-Biotic (CEI2013-MP-10). We also acknowledge the “Unidad de Excelencia Química Aplicada a Biomedicina y Medioambiente” (UGR).

The original Article has been corrected.

