# Supplementary material for: Synthesis of a thermoresponsive crosslinked MEO2MA polymer coating on microclusters of iron oxide nanoparticles
Source: Sci Rep. 2021 Feb 17;11:3947. doi: 10.1038/s41598-021-83608-z (PMC7889631; doi:10.1038/s41598-021-83608-z)
Supplement: Supplementary file 1 — Supplementary Information [file 41598_2021_83608_MOESM1_ESM.docx]

Electronic Supporting Information

Synthesis of a thermoresponsive crosslinked MEO_2_MA polymer coating on microclusters of iron oxide nanoparticles

Alejandro Lapresta-Fernández,^a,b*^ Alfonso Salinas-Castillo,^a,b^ and Luis Fermín Capitán-Vallvey^a,b^

^a^ ECsens group, Department of Analytical Chemistry, Campus Fuentenueva, University of Granada, 18071 Granada, Spain Fax: +34 958 243328; Tel: +34 958 248436; E-mail: lapresta@ugr.es

^b^ Unit of Excellence in Chemistry applied to Biomedicine and the Environment of the Uni-versity of Granada.

*Corresponding author: [lapresta@ugr.es](mailto:alejandro_lapresta@hotmail.com) Fax: +34 958 243328; Tel: +34 958 248436;

**Keywords:** precipitation polymerization, MEO_2_MA, thermoresponsive polymer, iron oxide nanoparticles,

**Figure S1.** Thermal collapse of the D_H_ of the hybrid Fe_3_O_4_@MEO_2_MA microgels when cooling from 49 to 9 ºC (green dots). Firstly, a heating from 9 to 49ºC was applied observing that the D_H_ follows a temperature dependence (sigmoidal trend, (red line and black squares)). However, when applying a subsequent cooling (from 49 to 1 ºC) the D_H_ did not show any trend, involving an irreversible behaviour of the hybrid microgels. Errors bars indicate the standard deviation of three replicates

**1.- DLS traces used in Figure 3a with their associated autocorrelation plots.** (*Pk 1 Mean Int* means the main intensity for the main peak).

| Temperature (ºC) | Pk 1 Mean Int | Average | Standard deviation |
| --- | --- | --- | --- |
| 9 | 348.3 | 377.0 | 61.9 |
|  | 334.7 |  |  |
|  | 448.1 |  |  |

| Temperature (ºC) | Pk 1 Mean Int | Average | Standard deviation |
| --- | --- | --- | --- |
| 13 | 270.4 | 366.6 | 88.7 |
|  | 445.1 |  |  |
|  | 384.3 |  |  |

| Temperature (ºC) | Pk 1 Mean Int | Average | Standard deviation |
| --- | --- | --- | --- |
| 17 | 236.2 | 282.4 | 46.4 |
|  | 282 |  |  |
|  | 328.9 |  |  |

| Temperature (ºC) | Pk 1 Mean Int | Average | Standard deviation |
| --- | --- | --- | --- |
| 21 | 174.8 | 269.5 | 83.1 |
|  | 330.2 |  |  |
|  | 303.6 |  |  |

| Temperature (ºC) | Pk 1 Mean Int | Average | Standard deviation |
| --- | --- | --- | --- |
| 25 | 163.5 | 218.4 | 51.6 |
|  | 226 |  |  |
|  | 265.8 |  |  |

| Temperature (ºC) | Pk 1 Mean Int | Average | Standard deviation |
| --- | --- | --- | --- |
| 29 | 160.9 | 173.4 | 17.7 |
|  | 185.9 |  |  |
|  | 350.2* |  |  |

*Data considered as anomalous. Pk 2 mean Int: 130.4

| Temperature (ºC) | Pk 1 Mean Int | Average | Standard deviation |
| --- | --- | --- | --- |
| 33.1 | 168.1 | 180.5 | 52.0 |
|  | 135.8 |  |  |
|  | 237.5 |  |  |

| Temperature (ºC) | Pk 1 Mean Int | Average | Standard deviation |
| --- | --- | --- | --- |
| 37 | 141.9 | 147.8 | 17.2 |
|  | 134.3 |  |  |
|  | 167.2 |  |  |

| Temperature (ºC) | Pk 1 Mean Int | Average | Standard deviation |
| --- | --- | --- | --- |
| 41 | 127.7 | 143.7 | 18.1 |
|  | 139.9 |  |  |
|  | 163.4 |  |  |

| Temperature (ºC) | Pk 1 Mean Int | Average | Standard deviation |
| --- | --- | --- | --- |
| 45 | 136.6 | 136.5 | 7.2 |
|  | 143.6 |  |  |
|  | 129.3 |  |  |

| Temperature (ºC) | Pk 1 Mean Int | Average | Standard deviation |
| --- | --- | --- | --- |
| 49 | 141.5 | 146.0 | 6.0 |
|  | 152.8 |  |  |
|  | 143.6 |  |  |

**2.- DLS traces obtained when cooling the hybrid microgels from 49 to 1 ºC (green dots in Figure S1). Data correspond to the irreversible behaviour in the D_H_ with Temperature.** (*Pk 1 Mean Int* means the main intensity for the main peak).

| Temperature (ºC) | Pk 1 Mean Int | Average | Standard deviation |
| --- | --- | --- | --- |
| 37 | 138.6 | 149.9 | 12.8 |
|  | 147.3 |  |  |
|  | 163.8 |  |  |

| Temperature (ºC) | Pk 1 Mean Int | Average | Standard deviation |
| --- | --- | --- | --- |
| 33 | 204.9 | 274.8 | 88.1 |
|  | 245.7 |  |  |
|  | 373.7 |  |  |

| Temperature (ºC) | Pk 1 Mean Int | Average | Standard deviation |
| --- | --- | --- | --- |
| 29 | 178.3 | 270.8 | 94.1 |
|  | 267.7 |  |  |
|  | 366.5 |  |  |

| Temperature (ºC) | Pk 1 Mean Int | Average | Standard deviation |
| --- | --- | --- | --- |
| 25 | 203.2 | 305.8 | 140.9 |
|  | 466.5 |  |  |
|  | 247.8 |  |  |

| Temperature (ºC) | Pk 1 Mean Int | Average | Standard deviation |
| --- | --- | --- | --- |
| 21 | 242.6 | 277.5 | 42.5 |
|  | 265.0 |  |  |
|  | 324.8 |  |  |

| Temperature (ºC) | Pk 1 Mean Int | Average | Standard deviation |
| --- | --- | --- | --- |
| 17 | 353.3 | 332.7 | 133 |
|  | 190.6 |  |  |
|  | 454.1 |  |  |

| Temperature (ºC) | Pk 1 Mean Int | Average | Standard deviation |
| --- | --- | --- | --- |
| 13 | 436.5 | 337.4 | 85.9 |
|  | 288.3 |  |  |
|  | 287.3 |  |  |

| Temperature (ºC) | Pk 1 Mean Int | Average | Standard deviation |
| --- | --- | --- | --- |
| 9 | 254.4 | 257.7 | 40.6 |
|  | 211.3 |  |  |
|  | 292.4 |  |  |

| Temperature (ºC) | Pk 1 Mean Int | Average | Standard deviation |
| --- | --- | --- | --- |
| 5 | 161.7 | 226.6 | 56.9 |
|  | 250.7 |  |  |
|  | 267.5 |  |  |

| Temperature (ºC) | Pk 1 Mean Int | Average | Standard deviation |
| --- | --- | --- | --- |
| 1 | 241.9 | 249.2 | 80.9 |
|  | 333.5 |  |  |
|  | 172.1 |  |  |
